# Lycopene overproduction and in situ extraction in organic-aqueous culture systems using a metabolically engineered *Escherichia coli*

**DOI:** 10.1186/s13568-015-0150-3

**Published:** 2015-09-22

**Authors:** Julia Gallego-Jara, Teresa de Diego, Álvaro del Real, Ana Écija-Conesa, Arturo Manjón, Manuel Cánovas

**Affiliations:** Department of Biochemistry and Molecular Biology (B) and Immunology, Faculty of Chemistry, University of Murcia, Campus de Espinardo, Regional Campus of International Excellence ‘‘Campus Mare Nostrum’’, P.O. Box 4021, 30100 Murcia, Spain

**Keywords:** Lycopene, Metabolically engineered *Escherichia coli*, Fed-batch culture, Organic-aqueous culture system

## Abstract

Lycopene is an import ant compound with an increasing industrial value. However, there is still no biotechnological process to obtain it. In this study, a semi-continuous system for lycopene extraction from recombinant *Escherichia coli BL*21 cells is proposed. A two-phase culture mode using organic solvents was found to maximize lycopene production through in situ extraction from cells. Within the reactor, three phases were formed during the process: an aqueous phase containing the recombinant *E. coli*, an interphase, and an organic phase. Lycopene was extracted from the cells to both the interphase and the organic phase and, consequently, thus enhancing its production. Maximum lycopene production (74.71 ± 3.74 mg L^−1^) was obtained for an octane-aqueous culture system using the *E. coli* BL21LF strain, a process that doubled the level obtained in the control aqueous culture. Study of the interphase by transmission electron microscopy (TEM) showed the proteo-lipidic nature and the high storage capacity of lycopene. Moreover, a cell viability test by flow cytometry (CF) after 24 h of culture indicated that 24 % of the population could be re-used. Therefore, a batch series reactor was designed for semi-continuous lycopene extraction. After five cycles of operation (120 h), lycopene production was similar to that obtained in the control aqueous medium. A final specific lycopene yield of up to 49.70 ± 2.48 mg g^−1^ was reached at 24 h, which represents to the highest titer to date. In conclusion, the aqueous-organic semi-continuous culture system proposed is the first designed for lycopene extraction, representing an important breakthrough in the development of a competitive biotechnological process for lycopene production and extraction.

**Electronic supplementary material:**

The online version of this article (doi:10.1186/s13568-015-0150-3) contains supplementary material, which is available to authorized users.

## Introduction

Lycopene is a tetraterpenoid (C40) precursor of carotenoids. Traditionally, it was considered a colorant and a food additive, but new applications have been proposed for use as an antioxidant (Chasse et al. 2001) and anticarcinogen (Giovannucci et al. 2002; Rabi and Gupta 2008) and for preventing against cardiovascular diseases (Rao 2002), hepatic fibro-genesis (Kitade et al. 2002) or human papillomavirus persistence (Sedjo et al. 2002). In spite of its great importance, most of lycopene is obtained from tomato, and no a competitive biotechnological exists process for its production.

In industry, methods based on metabolic engineering are the most profitable due to their high productivity and, consequently, the search for a biotechnological method for lycopene production is an important challenge for many researchers. In the last decade, many studies have been reported concerning lycopene production by metabolic engineering, some of which are based on *E. coli*, the most important cell factory microorganism in biotechnology (Kim and Keasling 2001; Martin et al. 2003; Alper et al. 2006; Yuan et al. 2006; Rodriguez-Villalon et al. 2008; Yoon et al. 2008; Zhou et al. 2012; Chen et al. 2013). Recombinant *E. coli* are capable of biosynthesizing lycopene through either the mevalonate (MEP) or the non-mevalonate route. Although the *E. coli* possesses the genes of the non-mevalonate route (or 2-*C*-methyl-d-erythritol 4-phosphate pathway) for isopentenyl pyrophosphate (IPP) synthesis, is still requires the following three enzymes, geranylgeranyl pyrophosphate (GGPP) synthase (*crt*E), phytoene synthase (*crt*B) and phytoene desaturase (*crt*I) to be able to synthesize lycopene. Therefore, a recombinant bacterium which contains these enzymes is necessary (Additional file 1: Figure S1). Previous works demonstrated that carotenoid production can be improved by increasing the amount of IPP and its isomer dimethylallyl pyrophosphate (DMAPP) amount available in the recombinant *E. coli* engineered (Jin and Stephanopoulos 2007; Zhou et al. 2012; Zhang et al. 2013). Besides to *E. coli*, carotenogenic microorganisms, such as *Blakeslea trispona* (Xu et al. 2007) and the non-carotenogenic yeasts, *Pichia pastoris* (Araya-Garay et al. 2012) and *Saccharomyces cerevesiae* (Bahieldin et al. 2014), have been used to produce lycopene.

Despite the achievements made to date, there is still no competitive biotechnological method to compete with lycopene extraction from tomatoes. The main problems of these metabolic engineering processes are plasmid instability and the low capability to accumulate lycopene in the cytoplasmic membrane from non-carotenogenic organisms (Wang et al. 2012). Hence, the in situ recovery of lycopene from a recombinant *E. coli* strain is the goal for achieving a competitive biotechnological process. To our knowledge there are few studies concerning lycopene extraction from *E. coli*. It has been reported an in situ process based on lycopene overproduction and recovery using octane and decane as extraction solvents (Yoon et al. 2008). However, the lycopene percentage extracted was quite low. The partial digestion of bacterial walls with lysozyme improved the system extractive capacity, although this digestion was quickly reverted as bacteria duplicated. In 2011, an in situ extraction process of retinoids from *E. coli* was reported (Jang et al. 2011), in which dodecane was used as extraction solvent, attaining a 68-fold higher productivity than attained with the aqueous system.

Hence, the search for a competitive system concerning both lycopene production and extraction is of great interest in the biotechnology field. In this paper, we propose the first semi-continuous system to produce and extract high amounts of lycopene employing a recombinant *E. coli* strain.

## Materials and methods

### Cell mass and specific growth rate

Cell mass was determined using a linear calibration curve relating optical density at 600 nm (OD600) and dry cell weight (R^2^ = 0.99). Cells were filtered and washed thoroughly with distilled water, and then dried at 130 °C for 24 h to a constant weight using a thermobalance (Electronic Moisture Analyzer model MA35, Sartorius). The exponential growth phase was identified and the specific growth rate was determined for all culture strains cultures (Sauer et al. 1999).

### Transformation and culture conditions

Chemically competent *E. coli* K12 (BW25113) (Baba et al. 2006) and BL21-Gold (DE3) (Agilent Technologies) cells were transformed with the pAC-Lyc plasmid, which contained three genes of the lycopene pathway, *crtE*, *crtB* and *crtI*, and a chloramphenicol resistance gene, by heat shock at 42 °C. The resulting strains were called *E. coli* K12L and *E. coli* BL21L, respectively. Then, *E. coli* BL21L was made competent again and co-transformed with the pET-SIDF and pET-SIDFG plasmids, obtaining the strains *E. coli* BL21LF and *E. coli* BL21LG, respectively (Table 1). These plasmids contained the genes *dxs, idi, ispD, ispF* (pET-SIDF) and *dxs, idi, ispD, ispF* and *ispG* (pET-SIDFG) and an expression plasmid controlled by the inducible promoter T7. Besides, they showed ampicillin resistance. The plasmids pAC-Lyc, pET-SIDF and pET-SIDFG were kindly supplied by Prof. G. Stephanopoulos (Department of Chemical Engineering, Institute of Technology, Cambridge, Massachusetts, EEUU) (Zhou et al. 2012).

**Table 1:** Recombinant *E. coli* cells used in this study

Strains	Plasmids			Named in this study
	pAC-Lyc	pET-SIDF	pET-SIDFG	
*E. coli* K12	X			*E. coli* K12L
*E. coli* BL21	X			*E. coli* BL21L
	X	X		*E. coli* BL21LF
	X		X	*E. coli* BL21LG

Lycopene biosynthesis was carried out in triplicate in 500 mL flasks containing 50 mL of MM9 medium with 20 mM glucose or 40 mM glycerol using an orbital shaking at 200 rpm and 28 °C. The culture medium was supplemented with appropriate antibiotics (30 μg mL^−1^ chloramphenicol and/or 100 μg mL^−1^ ampicillin).

### Lycopene extraction

Metabolically engineered *E. coli* cells were harvested by centrifugation at 10,000×*g* for 5 min at 4 °C. The cell pellet was resuspended in 1 mL of acetone and vigorously stirred for 10 min at 4 °C. The mixture was then centrifuged at 10,000×*g* for 10 min, and the acetone supernatant was filtered through a 0.2 μm nylon sterile filter. Then, samples were lyophilized (Thermo Scientific Heto PowerDry) and the final extract was resuspended in 0.1 mL of a 50:50 (v:v) mixture of A:B mobile phases for HPLC analysis. The lycopene extracted from the interphase was treated using the same procedure. The organic solvent phase was filtered through a 0.2 μm nylon sterile filter and lyophilized.

### Lycopene quantification by HPLC

The HPLC separation was performed on a Shimadzu HPLC equipped with a multi-channel pump (mod LC-20AD) and a DAD detector (mod SPD-M20A) with a Develosil^®^ C30-UG-5 column (250 mm × 4.6 mm × 5 μm) from Phenomenex. Elution conditions were based on the chromatographic method developed by (Sander et al. 1994) with modifications. Two mobile phases were used: phase A, composed of methanol and water (96:4, v:v) and phase B, tert-butyl methyl ether. The flow rate was 1.2 mL min^−1^ and the injection volume 40 μL. The column was thermostated at 30 °C. The separation of carotenoid standards and extracts was carried out using a linear mobile phase gradient from 50:50 (volume ratio, A/B) to 37:63 (volume ratio, A/B) in 12 min; then the system was restored to its initial condition for 5 min. The concentrations of lycopene, 13-*cis*-lycopene and phytoene were calculated using response factors relative to the internal standard, β-apo-8-carotenal. Carotenoid identification was carried out by comparing the retention times and absorption spectra characteristics (Additional file 1: Table S1) by reference to standards purchased from Sigma Aldrich. Detection was performed at 472 nm for lycopene and 13-*cis*-lycopene and at 285 nm for phytoene (Additional file 1: Figure S2). Measurements obtained from cell extracts were compared to curves generated from standards (R^2^ = 0.99).

### Transmission electron microscopy (TEM)

*Escherichia coli* BL21LF cells and interphase samples were fixed with 3 % glutaraldehyde for 30 min and prepared as previously described (Huxley and Zubay 1960). The ultrathin sections were cut in a Reichert–Young ultramicrotom. Staining was carried out with 2 % uranyl acetate. Sections were then examined by using a Carl Zeiss EM 10 C electron microscope.

### Flow cytometry (FCM)

Samples were run by flow cytometry (FCM) in a Becton–Dickinson FASort model equipped with an argon laser for excitation at 488 nm and 15 mW and filters at 525 and 630 nm. Samples were adjusted to an event rate of 800–2000 cells s^−1^ and a total of 10,000 events were registered per sample. To determine cell viability by FCM, double staining was performed accordingly to (Hewitt et al. 1999). PI and BOX were used for viability studies on living cells. The FCM probe fluoresceinpropidium iodide (PI) was purchased from Sigma–Aldrich, while bis-(1,3-dibutylbarbituric acid) trimethine oxonol (Bis-oxonol, BOX) was purchased from Molecular Probes Inc. Stained cells were diluted in phosphate buffered saline solution pH 7.2 (PBS). FALS and RALS values allowed cell debris discrimination and a total of 10,000 events were used for statistical data analysis.

Heat stressed cells treated at 60 °C for 30 min and exponentially growing cells were used as positive and negative controls, respectively. The green fluorescence channel for BOX-stained cells (X-axis) was plotted versus the red fluorescence channel for PI stained cells (Y-axis). Flow cytometry data were analysed with WinList 5.0 (Verity Software House) software.

## Results

### Optimization of IPTG concentration

**Fig. 1:**
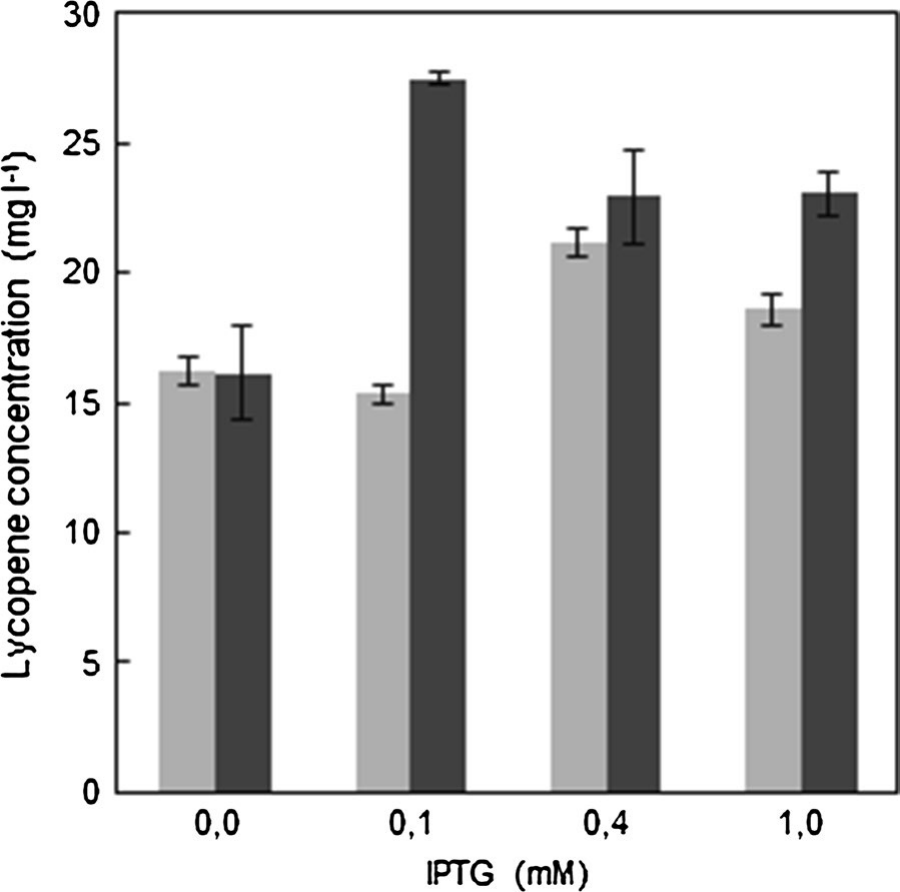
Lycopene production at different IPTG concentrations after 24 h in glucose culture. *Grey bars* and *solid bars* represent the results obtained using *E. coli* BL21F and the *E. coli* BL21G, respectively. Data represent the means and standard deviations from three separate experiments

Recombinant *E. coli* BL21LF and *E. coli* BL21LG (Table 1) were cultivated in MM9 medium containing 20 mM glucose as carbon source with 200 rpm orbital shaking and 28 °C. When the cultures reached 0.5 OD, the IPTG inductor was added at concentrations ranging from 0 to 1.0 mM. Samples were taken from each culture for lycopene extraction at 24 h, when the lycopene production was maximum. As shown in Fig. 1, a positive correlation between lycopene production and inductor concentration was noted for the two recombinant *E. coli* BL21 strains. Based on these results, the IPTG concentration selected was 0.1 and 0.4 mM for *E. coli* BL21LF and *E. coli* BL21LG, respectively.

### Optimization of culture conditions

In order to determine the optimal culture conditions, besides lycopene, the 13-*cis*-lycopene and phytoene content were quantified, the first since it is a lycopene isomer and is often produced by lycopene oxidative degradation (Chasse et al. 2001) and the second, as the precursor of lycopene. To select a carbon source for lycopene production, 50 mL batch cultures for the whole recombinant *E. coli* strains (K12L, BL21L, BL21LF and BL21LG) were carried out using either 40 mM glycerol or 20 mM glucose (Additional file 1: Figure S3). All cultures were made by orbital shaking at 200 rpm and 28 °C, which is the optimal temperature for lycopene biosynthesis (Kim et al. 2011). The biomass, lycopene, 13-*cis*-lycopene and phytoene content were determined at 24 h. The specific growth rate was also calculated for each culture (Table 2).

**Table 2:** Effect of carbon source on cell growth (OD), lycopene production and specific growth rate (μ_max_) in the *E. coli* aqueous cultures

Carbon source	OD (600 nm)	Lycopene (mg L^−1^)	μ_max_ (h^−1^)
Glucose			
*E. coli* K12L	3.56 ± 0.07	16.18 ± 0.81	0.33 ± 0.03
*E. coli* BL21L	3.86 ± 0.04	11.51 ± 0.52	0.43 ± 0.01
*E. coli* BL21LF	4.27 ± 0.04	27.51 ± 1.35	0.43 ± 0.02
*E. coli* BL21LG	3.29 ± 0.17	21.15 ± 1.01	0.35 ± 0.04
Glycerol			
*E. coli* K12L	3.61 ± 0.15	20.88 ± 1.04	0.27 ± 0.01
*E. coli* BL21L	3.95 ± 0.24	14.84 ± 0.74	0.30 ± 0.04
*E. coli* BL21LF	2.79 ± 0.04	37.56 ± 1.41	0.31 ± 0.01
*E. coli* BL21LG	1.55 ± 0.06	27.28 ± 0.81	0.28 ± 0.06

Data represent the means and standard deviations from three separate experiments

**Fig. 2:**
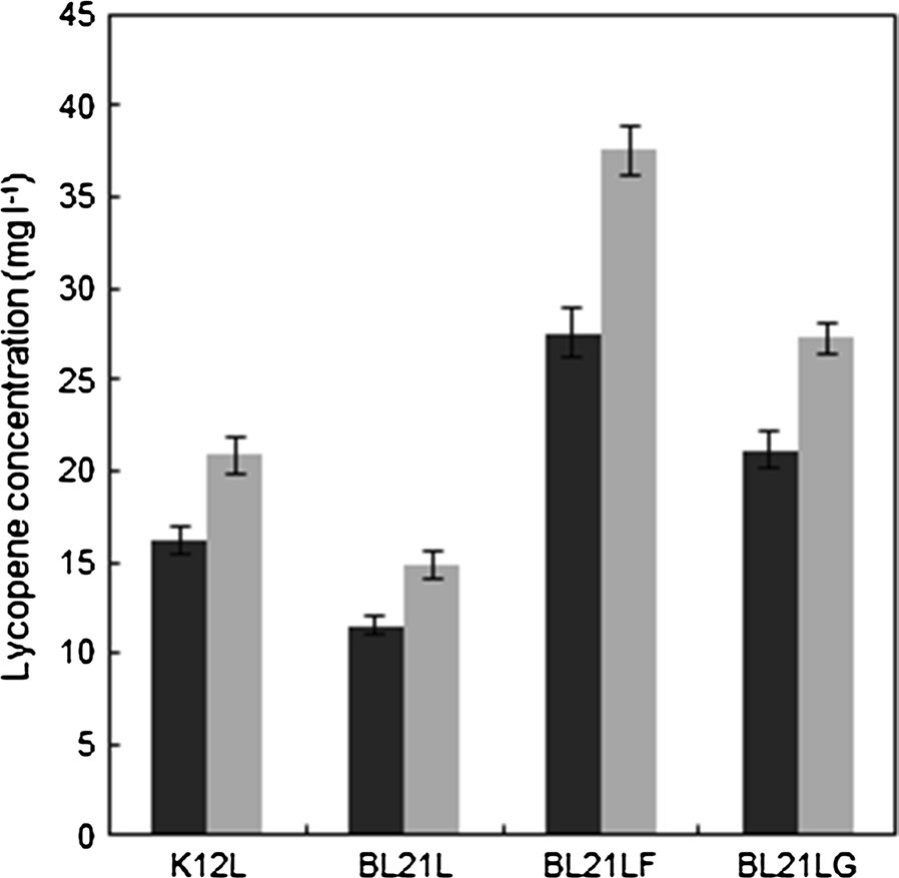
Comparison between glucose (*solid bars*) and glycerol (*grey bars*) as main carbon sources for lycopene production after 24 h using the recombinant *E. coli* strains. Data represent the means and standard deviations from three separate experiments

Glycerol cultures exhibited the highest lycopene production, whereas glucose cultures showed the highest cell mass and specific growth rate. In all cultures, the phytoene concentration was very low compared with lycopene (lower than 5 %), demonstrating a optimal expression of pAC-Lyc plasmid (Rodriguez-Villalon et al. 2008). The maximum lycopene content was obtained from the *Ecoli* BL21LF strain when glycerol was used as carbon source (Fig. 2). Therefore, glycerol was selected as carbon source for lycopene production with *E. coli* BL21LF in all subsequent studies.

The effect of key fermentation control parameters, such as shaking speed and light, on the production of lycopene were also tested. In the whole set of cultures exposed to light, the concentration of 13-*cis*-lycopene was very low compared with lycopene (lower than 8 %). When these same cultures were grown in darkness, no differences were detected for the isomer concentration, thus this additional precaution was discarded (data not shown). Orbital shaking speed was investigated as a putative bioreactor parameter responsible for controlling dissolved oxygen content and maximum cell density. Batch cultures in 500 mL flasks with 50 mL MM 9 medium were carried out at three orbital shaking speeds: 100, 200 and 400 rpm. However, there were no differences in lycopene production.

**Fig. 3:**
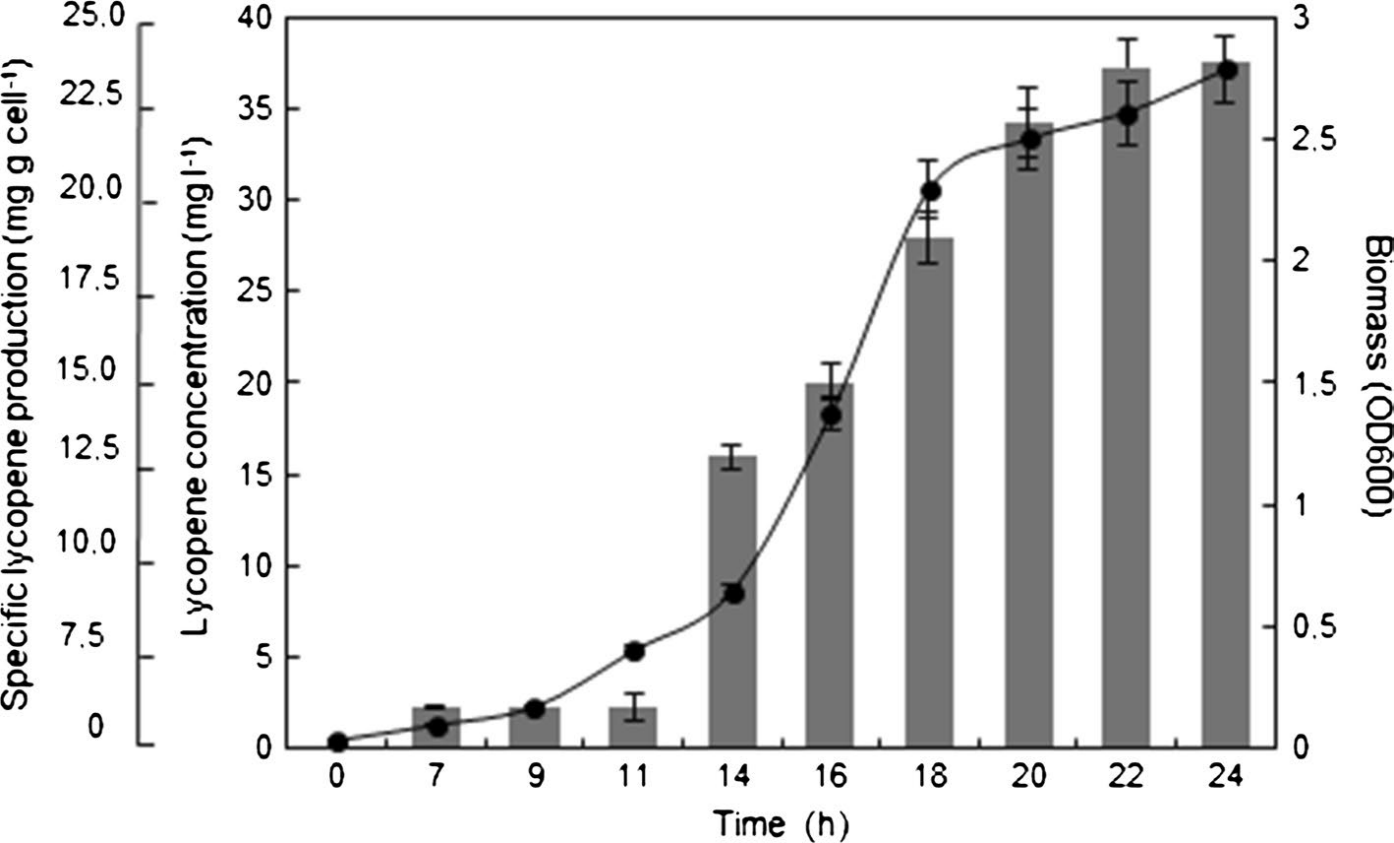
Specific lycopene content (*grey bars*) and biomass (*filled circle*) of recombinant *E. coli* BL21LF in 50 mL batch culture with 40 mM glycerol, 200 rpm and 28 °C. Data represent the means and standard deviations from three separate experiments

Lycopene production was evaluated in detail using glycerol as carbon source and the selected *E. coli* BL21LF in 50 mL batch culture and with 200 rpm orbital shaking at 28 °C. For this purpose, three samples were taken at different times up to 24 h (Fig. 3). Lycopene production started during the exponential growth phase, and the maximum lycopene concentration was reached in the stationary phase, since it is a secondary metabolite biomass dependent.

### Increase of lycopene production via in situ extraction in organic-aqueous culture systems

The effect of organic solvents on cell growth and lycopene production in metabolically engineered *E. coli* BL21LF was investigated in batch cultures. Organic solvents for lycopene extraction were selected as a function of the log P solvent, which ranged from 3.76, 4.27, 4.78, 5.8 to 6.31 for heptane, hexane, octane, decane, undecane and dodecane, respectively.

Batch cultures were performed using an organic:culture broth volume ratio of 1:5 (v/v) for the whole set of the 50 mL cultures. Organic solvent was added when the biomass reached 0.5 OD, in order not to affect cell growth. Hexane and heptane were rejected; hexane due to its high volatility and heptane as a consequence of its toxicity to cells. An aqueous culture without organic solvent was used as control system and the resulting lycopene production (37.56 ± 1.41 mg L^−1^) was used to normalize lycopene biosynthesis.

**Fig. 4:**
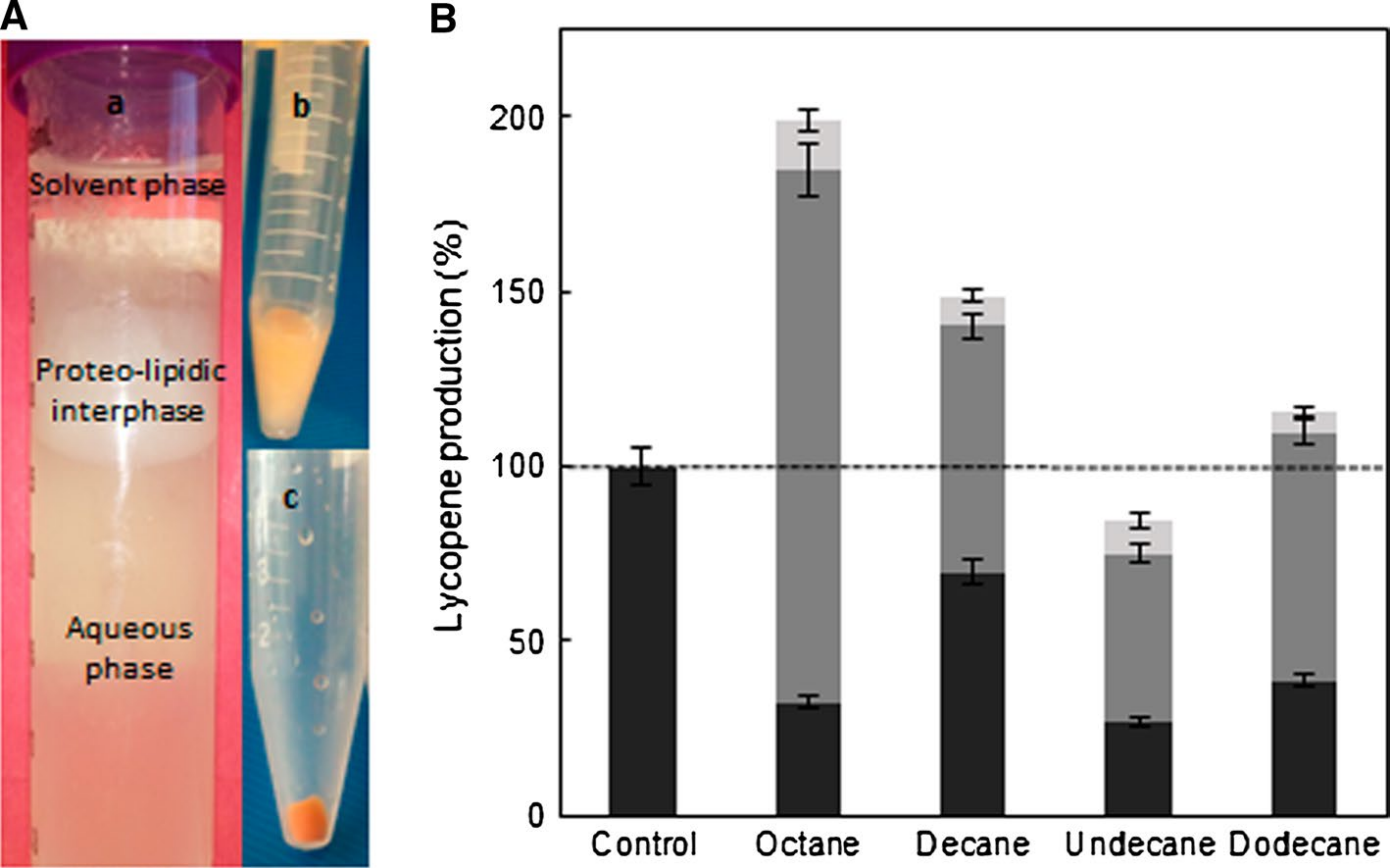
**A** Tri-phasic culture system for aqueous-organic systems. *b* Interphase. *c* *E. coli BL21LF* cells. Both phases were *orange* by the presence of lycopene. **B** Lycopene production normalized at 24 h for the organic-aqueous culture systems with *E. coli BL21LF*, distributed among three phases: aqueous phase containing *E. coli BL21LF* cells (*solid bars*), interphase (*grey bars*) and organic phase (*open bars*)

In all the aqueous-organic systems, three phases were visible after 24 h in the presence of organic solvent: an aqueous phase containing the *E. coli* BL21LF cells, an interphase and the organic phase. This tri-phasic culture system and normalized lycopene production at 24 h for all the organic-aqueous culture systems can be seen in Fig. 4A, B. Interestingly, lycopene production greatly increased with the addition of organic solvent except for undecane, although the lycopene production profile presented a negative correlation with the solvent log P. Final lycopene production after 24 h was 198.9, 148.6 and 115.97 % for octane, decane and dodecane, respectively, with respect to the control aqueous culture. In these organic-aqueous culture systems, lycopene was extracted from the cells to the interphase and the organic phase, which greatly enhanced production. Maximum lycopene production was obtained for the octane-aqueous system reaching 74.71 ± 3.74 mg L^−1^ (198.9 %). This lycopene production was distributed among the three phases formed: 12.24 ± 0.61 mg L^−1^ into *E. coli* BL21LF cells of the aqueous phase, 57.14 ± 2.85 mg L^−1^ in the interphase and 5.32 ± 0.24 mg L^−1^ in the octane phase. As regards to the cell density, the OD at 600 nm of the aqueous media was similar to the aqueous-organic systems at 24 h, around 4.00 ± 0.35. The *E. coli* BL21LF growth culture is shown in Additional file 1: Figure S4.

**Fig. 5:**
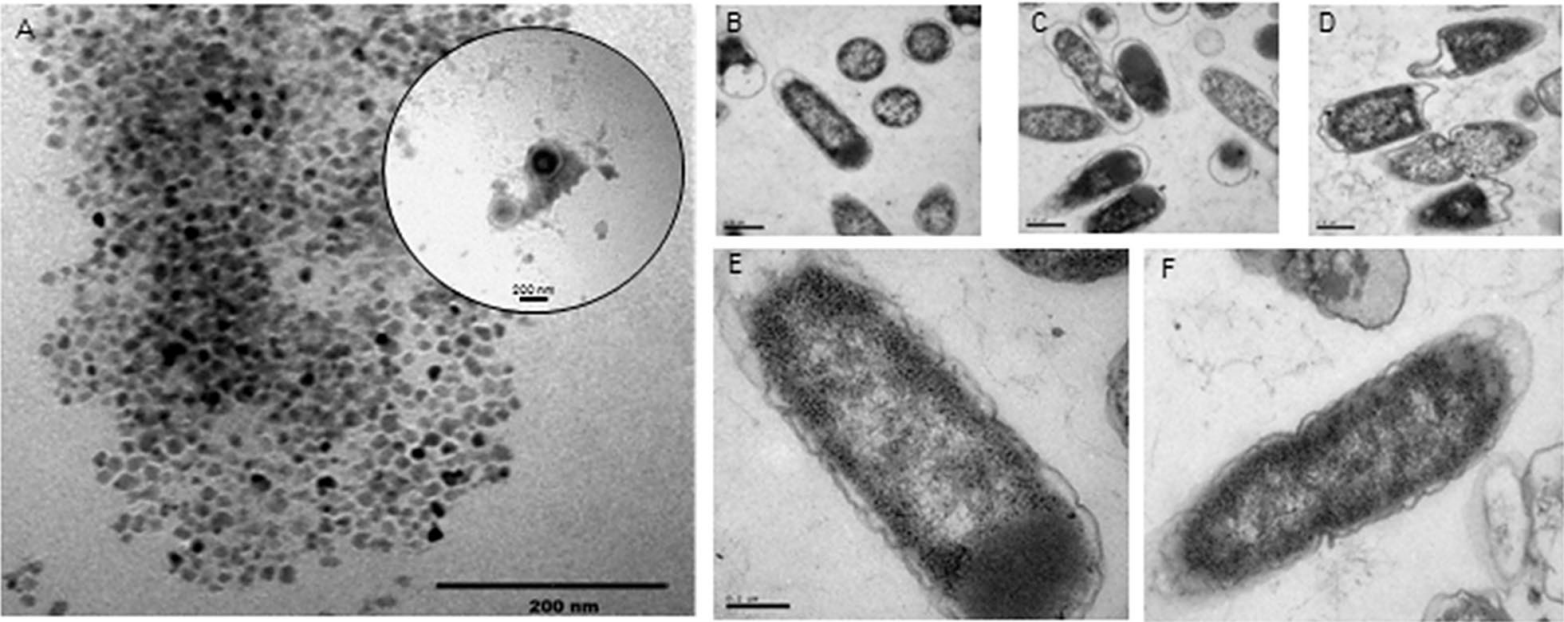
**A** Transmission electron microscopy of the interphase of the octane-aqueous culture prepared with negative staining. Transmission electron microscopy of *E. coli* BL21LF: **B** and **C** in aqueous media, **D** and **E** in aqueous-octane culture system and **F** in aqueous-heptane culture system. Amplifications were ×40,000 for **B**, **C** and **D** and ×100,000 for **E** and **F**

In order to study the physicochemical properties of the interphase formed in the aqueous-organic systems, transmission electron microscopy (TEM) was used (Fig. 5A). Spherical particles of homogeneous size ranging from 15 to 20 nm were observed, demonstrating its proteo-lipidic nature and with high lycopene storage capacity of these particles, representing 76.5 % (57.14 ± 2.85 mg L^−1^) of the total culture content. TEM was also used to visualize *E. coli* BL21LF cells after 24 h (Fig. 5B–F). Pictures b and c show *E. coli* BL21LF cells in the aqueous medium, whereas d and e show *E. coli* BL21F cell within the octane-aqueous culture system, and f shows *E. coli* BL21LF in the heptane-aqueous culture system. As depicted, *E. coli* BL21LF cell growth in aqueous media entirely maintained the outer membrane structure, and even the lipid double layer could be observed. But when the octane-aqueous culture system was employed, the cells partially lost the structural integrity of their outer membrane. This explains the high extractive capacity of octane for lycopene and the proteo-lipidic interphase formation. Cells completely lost their structural integrity when heptane was used.

**Fig. 6:**
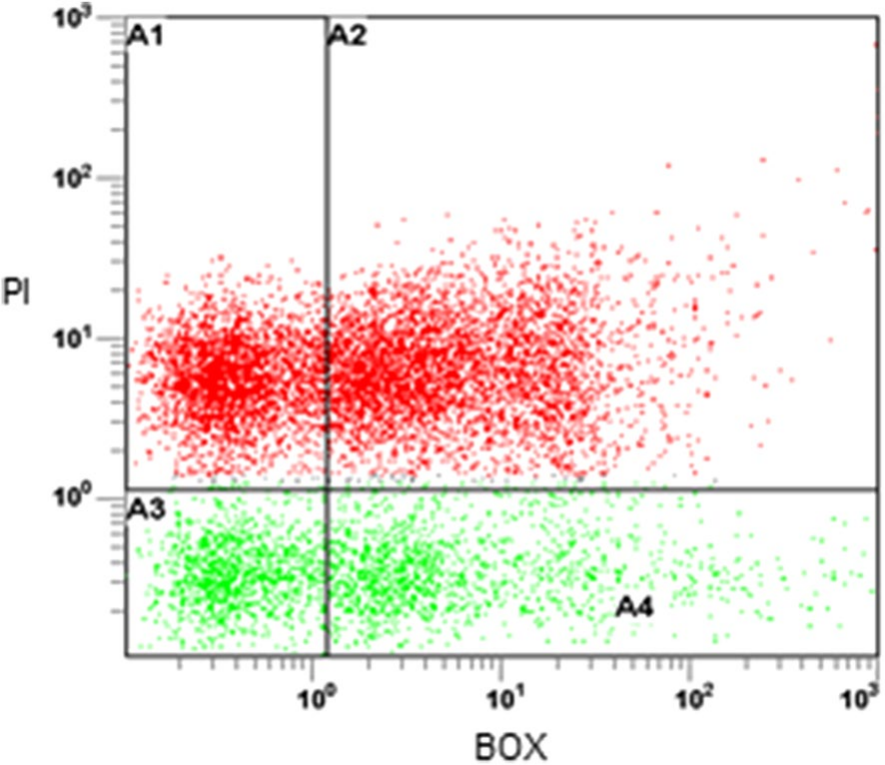
Flow cytometry (FCM) of the *E. coli* BL21LF strains in an aqueous-octane culture system. *Green* fluorescence of cells (axis X) due to BOX is plotted versus *red* fluorescence (axis Y) due to PI. Samples were taken from the batch reactor at 24 h

To determine cell viability, flow cytometry (FCM) using scattered light was chosen. Two fluorochromes were employed simultaneously: bis-(1,3-dibutylbarbituric acid) trimethine oxonol (Bis-oxonol, BOX) and propidium iodide (PI). BOX is a lipophilic anionic compound, which accumulates intracellularly when the cytoplasmic membrane is depolarised, while PI binds to DNA, but cannot cross an intact cytoplasmic membrane (Hewitt et al. 1999). Cell analysis by FCM demonstrated that, during a fed-batch culture in an aqueous-octane system, there was a gradual change in the physiological state of *E. coli* BL21LF. From samples taken at 24 h, three main sub-populations of cells were observed (Fig. 6). These populations corresponded to healthy unstained cells (A3); cells with a depolarised cytoplasmic membrane (A4), stained with BOX; and dead cells with permeabilised membranes, namely cells stained with both PI and BOX (A2). From Fig. 6, the cell number and the percentage within each quadrant was determined using the CF software. The results are shown in Table 3. Cells from a microbial culture can be grouped according to their different metabolic states and/or extent of cell integrity: (a) intact and metabolically active cells, showing growth capacity, (b) depolarized cells, unable to maintain their intact membrane potential, but that can be recovered temporarily (Cánovas et al. 2007), (c) and dead cells with permeabilised and depolarised membrane (Hewitt et al. 1999). The aqueous-octane culture system *E. coli* BL21LF after 24 h, resulted in an 11.5 % of healthy unstained cells and a 13.24 % of deporalised but recoverable BOX stained cells. Therefore, both of them (24.74 %) can be considered viable for the continuous production of lycopene.

**Table 3:** Cells number and percentage in each one of the FCM windows

Window	Cells number	Percentage (%)
A1	179	1.79
A2	7347	73.47
A3	1150	11.50
A4	1324	13.24

Cell debris was identified on the basis of the FALS and RALS values

### Aqueous-octane culture system for in situ extraction and semi-continuous lycopene production

**Fig. 7:**
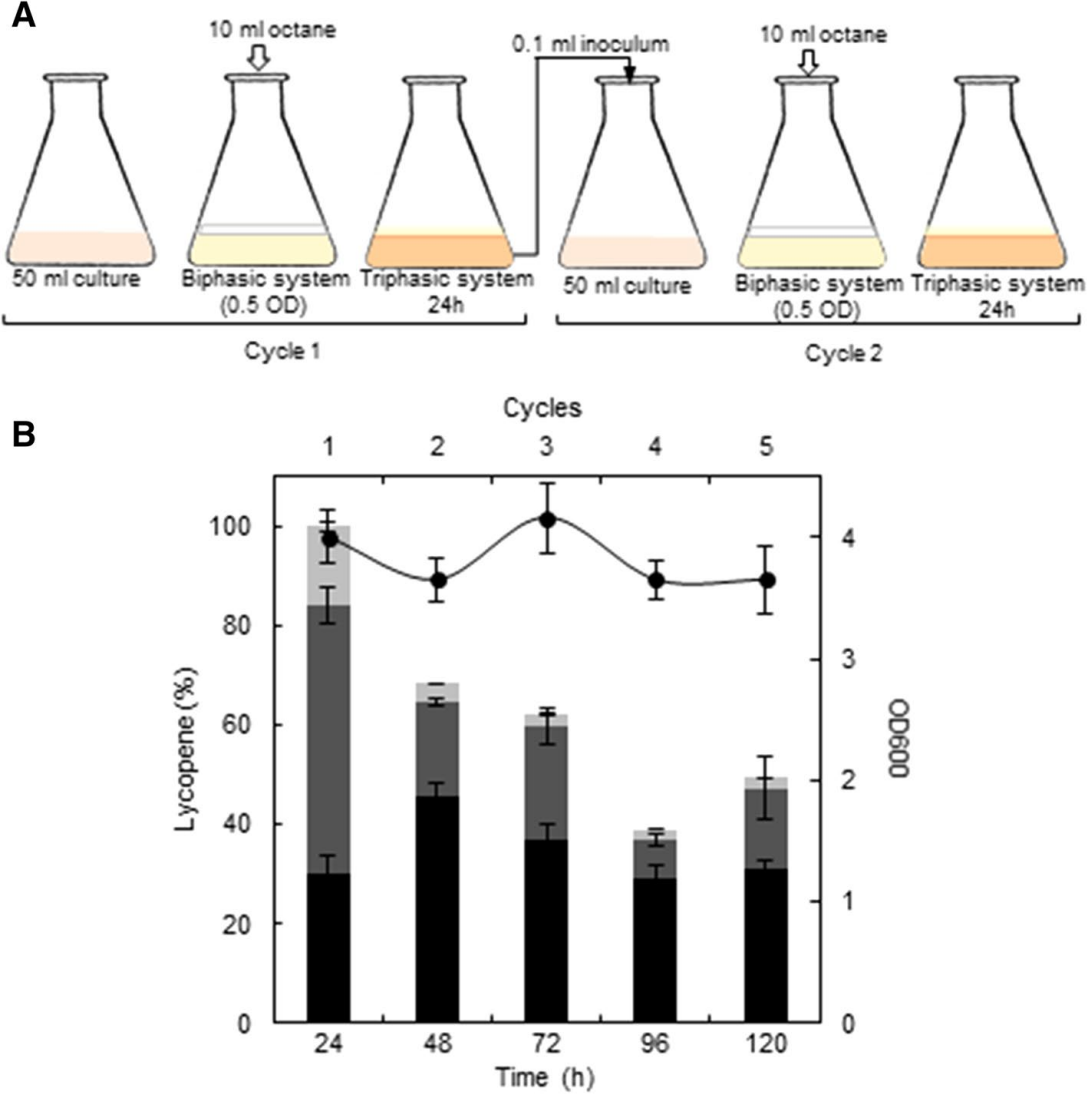
**a** Series batch reactor for lycopene over-production using *E. coli* BL21LF in an aqueous-octane culture system. **b** Normalized lycopene production distributed within each phase for each cycle: aqueous phase containing *E. coli BL21LF* cells (*solid bars*), interphase (*grey bars*) and organic phase (*light grey bars*), and biomass (*filled circle*). Data represent the means and standard deviations from three separate experiments

Maximum lycopene production was obtained from the octane-aqueous system (1:5 volume ratio) after 24 h of culture, when cell growth had ceased and the lycopene content was distributed among the three phases. From these results, a series batch reactor was designed for lycopene extraction, as depicted in Fig. 7a. The aqueous-octane culture system was maintained in operation for 24 h, then the volume of the aqueous phase corresponding to a final 0.05 OD was used as the inoculum for a second batch reactor (taking into account the viable cells fraction). This process was repeated 5 times.

Figure 7b displays the lycopene production distributed in each phase and cycle. The lycopene produced in the first cycle was used to normalize the lycopene content of the remaining cycles. In the first three, the lycopene production was 74.71 ± 3.74 mg L^−1^, 51.03 ± 2.51 mg L^−1^ and 46.45 ± 2.13 mg L^−1^, respectively, all of them higher than that obtained in the aqueous medium (37.56 ± 1.41 mg L^−1^). Lycopene production was 29.01 ± 1.52 mg L^−1^ and 36.96 ± 1.82 mg L^−1^ for the fourth and fifth cycle, respectively, similar to that obtained in the aqueous medium. The biomass reached in each batch reactor was similar, about 3.82 ± 0.35 OD, although production deceased with each cycle.

## Discussion

Metabolic engineering to increase lycopene production in *E. coli* has previously focused on overexpression of the several key isoprenoid genes (Alper et al. 2005). Hence, the first part of this study was focused on the optimization of both the recombinant *E. coli* strain and culture conditions. When the recombinant *E. coli* BL21LF and *E. coli* BL21G strains were used in combination with the IPTG-induced, lycopene production increased, although excessive IPTG concentration reduced lycopene biosynthesis (Fig. 1). This finding is consistent with previous observations of the induction inhibitory effects of lycopene production (Kim and Keasling 2001; Rodriguez-Villalon et al. 2008; Yoon et al. 2008) since, a high induction could cause a shortage of the available precursors for the essential metabolic roles. The IPTG concentrations selected were 0.1 and 0.4 mM for *E. coli* BL21LF and *E. coli* BL21LG, respectively. The lower lycopene production obtained with *E. coli* BL21LG was probably due to the high energy cost of the extra *isp*G gene overexpression, which encodes the HDS protein. It has been revealed that *ispG* gene basal expression is higher than the expression of other of isoprenoid genes under normal growth conditions (Yuan et al. 2006). Accordingly, a high multi-copy expression vector may cause a metabolic imbalance. In addition, the HDS enzyme (4-hydroxy-2-methyl-2-butenyl 4-diphosphate synthase) flavodoxin reductase and NADPH dependent metalloprotein, (Rohdich et al. 2003; Seemann et al. 2006) involve an extra expense of reducing power to carry out their catalytic functions (Hunter 2007). These assumptions could explain the *E. coli* BL21LG strain delay metabolic.

In previous studies, glycerol and glucose were compared as carbon sources for secondary metabolite production, glycerol being seen as a better carbon source than glucose (Lee 1996; Martin et al. 2001; Yoon et al. 2009). Glycerol reduces cell growth, but stimulates metabolite production (Fang and Demain 1997).

On the other hand, the recombinant *E. coli* BL21 and K12 strains showed differences in lycopene production, the latter producing 1.4-fold more than the *E. coli* BL21L strain (Fig. 2). Significant differences have been demonstrated at gene transcription and metabolomic profile levels between both *E. coli* strains (Alper et al. 2006). In addition, the outer membranes of *E. coli* K12 and *E. coli* BL21 also show differences in lipoprotein and lipid composition, which may influence cell envelope permeability and integrity. These differences could affect potential lycopene accumulation (Yoon et al. 2012; Marisch et al. 2013).

The maximum lycopene production and specific lycopene production were 37.56 ± 1.41 mg L^−1^ and 25.34 ± 1.2 mg g cell^−1^, respectively, which was obtained during stationary growth phase using *E. coli* BL21LF in 40 mM glycerol and 0.1 mM IPTG (Fig. 2). This production is excellent compared with the lycopene levels previously reported. Stephanopoulos group’s (2006) created a triple knockout strain, Δ*gdh*A Δ*ace*E Δ*fdh*F, which exhibited a lycopene production of 8.15 mg g cell^−1^ (Alper et al. 2006). Kin et al. (2011) used a metabolically engineered *E. coli* strain, reporting a lycopene production of 32 mg g cell^−1^ in fed-batch cultures with glycerol supplemented with glucose as auxiliary carbon source. Recently, a higher level of lycopene production (33.43 mg g cell^−1^) was attained by native appY promoter replacement of a T5 promoter, and the deletion of the *icl*R gene in *E. coli* CBW 12241 (Chen et al. 2013). High lycopene production was also achieved (18.49 mg g cell^−1^) using a CRP engineering strategy (Huang et al. 2015).

One of the main limitations of biotechnological lycopene production is the fact that it is stored as an intracellular product in the membrane (Fraser and Sandmann 1992). Therefore, special must be taken into account to identify the optimal parameters for continuous lycopene production. It has been assumed that the upper limit for the carotenoid production in a non-carotenogenic *E. coli* is around 2 mg g cell^−1^ due to the limited lipophilic carotenoid storage capacity of the membrane (Albrecht et al. 1999; Sandmann 2001). Hence, a new strategy is needed to overcome the lycopene accumulation barrier. Optimal parameters must be determined in order to promote lycopene production during both the exponential and stationary phase, while stimulating in situ extraction to prevent accumulation in the cell membrane. Moreover, when the whole cells are employed as biocatalyst, productivity may decrease due to end-product inhibition or accumulation: however, if the product is continuously removed by a solvent phase, an increase in activity/productivity can be attained. To achieve this aim, a two-phase culture system using an organic solvent was proposed to maximize the lycopene production through in situ extraction from the cells. Few studies using organic solvents for terpenes extraction have been published. It has been reported a two-phase culture system with dodecane for retinoids extraction using a metabolically engineered *E. coli* (Jang et al. 2011). In a previous report, a two-phase culture system with decane and 0.1 % (w v^−1^) Span 20 was successfully applied for lycopene production (9.6 ± 1.0 mg g^−1^) (Yoon et al. 2008). However, lycopene was inefficiently extracted from the recombinant *E. coli* strain without partial digestion of the cell wall with lysozyme. The authors used *E. coli* spheroplasts in order to increase the extraction, but their instability reduced the possibility of designing a continuous system.

Another aspect to consider in a biphasic system is the organic solvent toxicity toward microorganisms. This toxicity depends on its inherent toxicity and the intrinsic tolerance of the bacterial species and strains (Ramos et al. 2002). The toxicity of a solvent correlates with the logarithm of its partition coefficient in n-octanol and water (log P), meaning that, organic solvents with a log P of between 1.5 and 4.0 are toxic for microorganisms. Six organic solvents were selected for lycopene extraction as a function of the log P, ranging from 3.76 to 6.31. In all aqueous-organic systems tested in this study three phases were formed after 2 h of culture: an aqueous phase containing cells, an interphase and an organic phase (Fig. 4A). Lycopene was removed from the cells to the interphase and the organic phase, thus enhancing production. Maximum lycopene production was obtained from octane-aqueous systems (5:1, v v^−1^) (74.71 ± 3.74 mg L^−1^ or 49.70 ± 2.48 mg g cell^−1^), a 2-fold improvement over that attained in aqueous culture. This production was also much higher than that obtained from tomato, 0.42 mg g^−1^, which suggest it is a promising strategy for its industrial production (Sharma and Le Maguer 1996). This lycopene production rate is, to our knowledge, the highest reported in the literature to date. Moreover, the proteo-lipidic nature of the interphase demonstrated by TEM (Fig. 5A), showed a high lycopene storage capacity of 76.5 % (57.15 ± 2.86 mg L^−1^) with respect to the total production of lycopene in aqueous-octane systems (Fig. 4B). This interface was formed from partial outer membrane disintegration, while cells with structural integrity were found in the aqueous-octane systems after 24 h (Fig. 5D, E). Additionally, flow cytometry analysis of *E. coli* BL21LF cells showed a significant percentage (24.74 %) of viable and cultivatable cells for continuous lycopene production (Fig. 6; Table 3). From these results, a series batch reactor for semi-continuous lycopene extraction was designed (Fig. 7a). The biomass reached was similar for the all cycles, although lycopene production decreased with each of cycle. The results further support the idea that cell depolarisation indicates a decline in cell functionality due to energy depletion, but does not involve cell death. Besides, lycopene accumulation in the cell membrane seems to affect lycopene biosynthesis, since the amount of lycopene extracted from cells to the interphase and the organic phase decreased in each cycle. Nevertheless, the lycopene production obtained from the fifth cycle (120 h) was 36.96 ± 1.82 mg L^−1^, similar to that obtained in the aqueous medium (Fig. 7b).

In this study, semi-continuous lycopene overproduction and in situ extraction using a metabolically engineered *E. coli* strain is attained for the first time with an octane-aqueous culture system (1:5 volume ratio). In the future, we hope these findings will be useful for industry and constitute an important step forward in the development of a competitive biotechnological lycopene production system.

## Additional file

**Additional file 1: Table S1.** Retention times and absorption spectra characteristics of carotenoids. **Figure S1.** Biosynthetic pathway of lycopene in *Escherichia coli* from a native 2-C-methyl-D-erythritol 4-phosphate pathway (non-mevalonate pathway). Gene names and its encoded enzymes follow: *dxr* DXP reductoisomerase, *dxs* DXP synthase, *idi* IPP isomerase, *ispA* FPP synthase, *crtE* GGPP synthase, *crtB* phytoene synthase, *crtI* phytoene desaturase. **Figure S2.** Chromatogram of reference standards for 20 min measured at 472 and 285 nm, respectively. **Figure S4.** *E. coli* BL21 LF growth in the presence of octane during 24 h.

## References

[CR1] Albrecht M, Misawa N, Sandmann G (1999). Metabolic engineering of the terpenoid biosynthetic pathway of *Escherichia coli* for production of the carotenoids beta-carotene and zeaxanthin. Biotechnol Lett.

[CR2] Alper H, Miyaoku K, Stephanopoulos G (2005). Construction of lycopene-overproducing *E. coli* strains by combining systematic and combinatorial gene knockout targets. Nat Biotechnol.

[CR3] Alper H, Miyaoku K, Stephanopoulos G (2006). Characterization of lycopene-overproducing *E. coli* strains in high cell density fermentations. Appl Microbiol Biotechnol.

[CR4] Araya-Garay JM, Feijoo-Siota L, Rosa-Dos-Santos F, Veiga-Crespo P, Villa TG (2012). Construction of new *Pichia pastoris* X-33 strains for production of lycopene and beta-carotene. Appl Microbiol Biotechnol.

[CR5] Baba T, Ara T, Hasegawa M, Takai Y, Okumura Y, Baba M, Datsenko KA, Tomita M, Wanner BL, Mori H (2006). Construction of *Escherichia coli* K-12 in-frame, single-gene knockout mutants: the Keio collection. Mol Syst Biol.

[CR6] Bahieldin A, Gadalla NO, Al-Garni SM, Almehdar H, Noor S, Hassan SM, Shokry AM, Sabir JSM, Murata N (2014). Efficient production of lycopene in *Saccharomyces cerevisiae* by expression of synthetic crt genes from a plasmid harboring the ADH2 promoter. Plasmid.

[CR7] Cánovas M, García V, Bernal V, Torroglosa T, Iborra JL (2007). Analysis of *Escherichia coli* cell state by flow cytometry during whole cell catalyzed biotransformation for l-carnitine production. Process Biochem.

[CR8] Chasse G, Mak ML, Deretey E, Farkas I, Torday LL, Papp JG, Sarma DSR, Agarwal A, Chakravarthi S, Agarwal S, Rao AV (2001). An ab initio computational study on selected lycopene isomers. J Mol Struct THEOCHEM.

[CR9] Chen Y-Y, Shen H-J, Cui Y-Y, Chen S-G, Weng Z-M, Zhao M, Liu J-Z (2013). Chromosomal evolution of *Escherichia coli* for the efficient production of lycopene. BMC Biotechnol.

[CR10] Fang A, Demain A (1997). Influence of aeration and carbon source on production of microcin B17 by *Escherichia coli* ZK650. Appl Microbiol Biotechnol.

[CR11] Fraser PD, Sandmann G (1992). In vitro assays of three carotenogenic membrane-bound enzymes from *Escherichia coli* transformed with different crt genes. Biochem Biophys Res Commun.

[CR12] Giovannucci E, Rimm EB, Liu Y, Stampfer MJ, Willett WC (2002). A prospective study of tomato products, lycopene, and prostate cancer risk. J Natl Cancer Inst.

[CR13] Hewitt CJ, Nebe-Von Caron G, Nienow AW, McFarlane CM (1999). Use of multi-staining flow cytometry to characterise the physiological state of *Escherichia coli* W3110 in high cell density fed-batch cultures. Biotechnol Bioeng.

[CR14] Huang L, Pu Y, Yang X, Zhu X, Cai J, Xu Z (2015). Engineering of global regulator cAMP receptor protein (CRP) in *Escherichia coli* for improved lycopene production. J Biotechnol.

[CR15] Hunter WN (2007). The non-mevalonate pathway of isoprenoid precursor biosynthesis. J Biol Chem.

[CR16] Huxley H, Zubay G (1960). Electron microscope observations on the structure of microsomal particles from *E. coli*. J Mol Biol.

[CR17] Jang H-J, Yoon S-H, Ryu H-K, Kim J-H, Wang C-L, Kim J-Y, Oh D-K, Kim S-W (2011). Retinoid production using metabolically engineered *Escherichia coli* with a two-phase culture system. Microb Cell Fact.

[CR18] Jin YS, Stephanopoulos G (2007). Multi-dimensional gene target search for improving lycopene biosynthesis in *Escherichia coli*. Metab Eng.

[CR19] Kim S, Keasling JD (2001). Nonmevalonate isopentenyl diphosphate synthesis pathway in *Escherichia coli* enhances lycopene production. Biotechnol Bioeng.

[CR20] Kim YS, Lee JH, Kim NH, Yeom SJ, Kim SW, Oh DK (2011). Increase of lycopene production by supplementing auxiliary carbon sources in metabolically engineered *Escherichia coli*. Appl Microbiol Biotechnol.

[CR21] Kitade Y, Watanabe S, Masaki T, Nishioka M, Nishino H (2002). Inhibition of liver fibrosis in LEC rats by a carotenoid, lycopene, or a herbal medicine, Sho-saiko-to. Hepatol Res.

[CR22] Lee SY (1996). High cell-density culture of *Escherichia coli*. Trends Biotechnol.

[CR23] Marisch K, Bayer K, Scharl T, Mairhofer J, Krempl PM, Hummel K, Razzazi-Fazeli E, Striedner G (2013). A comparative analysis of industrial *Escherichia coli* K-12 and B strains in high-glucose batch cultivations on process-, transcriptome- and proteome level. PLoS One.

[CR24] Martin VJJ, Yoshikuni Y, Keasling JD (2001). The in vivo synthesis of plant sesquiterpenes by *Escherichia coli*. Biotechnol Bioeng.

[CR25] Martin VJJ, Pitera DJ, Withers ST, Newman JD, Keasling JD (2003). Engineering a mevalonate pathway in *Escherichia coli* for production of terpenoids. Nat Biotechnol.

[CR26] Rabi T, Gupta S (2008). Dietary terpenoids and prostate cancer chemoprevention. Front Biosci.

[CR27] Ramos JL, Duque E, Gallegos M-T, Godoy P, Ramos-Gonzalez MI, Rojas A, Teran W, Segura A (2002). Mechanisms of solvent tolerance in gram-negative bacteria. Annu Rev Microbiol.

[CR28] Rao A (2002). Lycopene, tomatoes, and the prevention of coronary heart disease. Exp Biol Med.

[CR29] Rodriguez-Villalon A, Perez-Gil J, Rodriguez-Concepcion M (2008). Carotenoid accumulation in bacteria with enhanced supply of isoprenoid precursors by upregulation of exogenous or endogenous pathways. J Biotechnol.

[CR30] Rohdich F, Zepeck F, Adam P, Hecht S, Kaiser J, Laupitz R, Gräwert T, Amslinger S, Eisenreich W, Bacher A, Arigoni D (2003). The deoxyxylulose phosphate pathway of isoprenoid biosynthesis: studies on the mechanisms of the reactions catalyzed by IspG and IspH protein. Proc Natl Acad Sci USA.

[CR31] Sander LC, Sharpless KE, Craft NE, Wise SA (1994). Development of engineered stationary phases for the separation of carotenoid isomers. Anal Chem.

[CR32] Sandmann G (2001). Carotenoid biosynthesis and biotechnological application. Arch Biochem Biophys.

[CR33] Sauer U, Lasko DR, Fiaux J, Hochuli M, Glaser R, Szyperski T, Wüthrich K, Bailey JE (1999). Metabolic flux ratio analysis of genetic and environmental modulations of *Escherichia coli* central carbon metabolism. J Bacteriol.

[CR34] Sedjo RL, Roe DJ, Abrahamsen M, Harris RB, Craft N, Baldwin S, Giuliano AR (2002) Vitamin A, carotenoids, and risk of persistent oncogenic human papillomavirus infection. Cancer Epidem Biomarkers Prev 11:876–884 12223432

[CR35] Seemann M, Tse Sum Bui B, Wolff M, Miginiac-Maslow M, Rohmer M (2006). Isoprenoid biosynthesis in plant chloroplasts via the MEP pathway: direct thylakoid/ferredoxin-dependent photoreduction of GcpE/IspG. FEBS Lett.

[CR36] Sharma SK, Le Maguer M (1996). Kinetics of lycopene degradation in tomato pulp solids under different processing and storage conditions. Food Res Int.

[CR37] Wang GS, Grammel H, Abou-Aisha K, Sägesser R, Ghosh R (2012). High-level production of the industrial product Lycopene by the photosynthetic Bacterium *Rhodospirillum rubrum*. Appl Environ Microbiol.

[CR38] Xu F, Yuan QP, Zhu Y (2007). Improved production of lycopene and b-carotene by *Blakeslea trispora* with oxygen-vectors. Process Biochem.

[CR39] Yoon K-W, Doo E-H, Kim S-W, Park J-B (2008). In situ recovery of lycopene during biosynthesis with recombinant *Escherichia coli*. J Biotechnol.

[CR40] Yoon SH, Lee SH, Das A, Ryu HK, Jang HJ, Kim JY, Oh DK, Keasling JD, Kim SW (2009). Combinatorial expression of bacterial whole mevalonate pathway for the production of β-carotene in *E. coli*. J Biotechnol.

[CR41] Yoon SH, Han M-J, Jeong H, Lee CH, Xia X-X, Lee D-H, Shim JH, Lee SY, Oh TK, Kim JF (2012). Comparative multi-omics systems analysis of *Escherichia coli* strains B and K-12. Genome Biol.

[CR42] Yuan LZ, Rouvière PE, LaRossa RA, Suh W (2006). Chromosomal promoter replacement of the isoprenoid pathway for enhancing carotenoid production in *E. coli*. Metab Eng.

[CR43] Zhang C, Chen X, Zou R, Zhou K, Stephanopoulos G, Too HP (2013). Combining genotype improvement and statistical media optimization for isoprenoid production in *E. coli*. PLoS One.

[CR44] Zhou K, Zou R, Stephanopoulos G, Too HP (2012). Metabolite profiling identified methylerythritol cyclodiphosphate efflux as a limiting step in microbial isoprenoid production. PLoS One.

