# Supplementary material for: Lycopene overproduction and in situ extraction in organic-aqueous culture systems using a metabolically engineered Escherichia coli
Source: AMB Express. 2015 Sep 22;5:65. doi: 10.1186/s13568-015-0150-3 (PMC4579157; doi:10.1186/s13568-015-0150-3)
Supplement: Supplementary file 1 — Additional file 1: Table S1. Retention times and absorption spectra characteristics of carotenoids. Figure S1. Biosynthetic pathway of lycopene in Escherichia coli from a native 2-C-methyl-D-erythritol 4-phosphate pathway (non-mevalonate pathway). Gene names and its encoded enzymes follow: dxr DXP reductoisomerase, dxs DXP synthase, idi IPP isomerase, ispA FPP synthase, crtE GGPP synthase, crtB phytoene synthase, crtI phytoene desaturase. Figure S2. Chromatogram of reference standards for 20 min measured at 472 and 285 nm, respectively. Figure S4. E. coli BL21 LF growth in the presence of octane during 24 h. [file 13568_2015_150_MOESM1_ESM.docx]

AMB Express

BIOTECHNOLOGICAL PRODUCTS AND PROCESS ENGINEERING

Supplementary Table and Figures for

**Lycopene overproduction and *in situ* extraction in organic-aqueous culture systems using a metabolically engineered *Escherichia coli***

Julia Gallego-Jara*, Teresa de Diego*, Álvaro del Real, Ana Écija-Conesa, Arturo Manjón, and Manuel Cánovas.

*Equally contributing authors

Department of Biochemistry and Molecular Biology (B) and Immunology, Faculty of Chemistry, University of Murcia, Campus de Espinardo, Regional Campus of International Excellence ‘‘Campus Mare Nostrum’’, P.O. Box 4021, Murcia E-30100, Spain.

Correspondence author: Teresa de Diego; E-mail: [tdp@um.es](mailto:tdp@um.es); phone 34-868 88 73 95 and fax number 34-868 88 41 48

This PDF file includes:

- Table S1
- Figures S1 to S3

**Table S1** Retention times and absorption spectra characteristics of carotenoids

| Peak  HPLC | Carotenoid | Tr (min) | λ (nm)  experimental | λ (nm)  theoretical | References |
| --- | --- | --- | --- | --- | --- |
| 1 | phytoene | 7.79 | (276) 285 (296) | (276) 286 (296.2) | (Sander et al. 1994) |
| 2 | (*13 cis*)-Lycopene | 8.93 | 297 360 439 466 495 | 297 360.4 439 465 495.5 | (Schierle et al. 1997) |
| 3 | (*all trans*)-Lycopene | 10.12 | 294 360 445 471 502 | 295 360.0 445 472 502.0 | (Schierle et al. 1997) |

**Fig. S1** Biosynthetic pathway of lycopene in *Escherichia coli* from a native 2-C-methyl-D-erythritol 4-phosphate pathway (non-mevalonate pathway). Gene names and its encoded enzymes follow: *dxr* DXP reductoisomerase, *dxs* DXP synthase, *idi* IPP isomerase, *ispA* FPP synthase, *crtE* GGPP synthase, *crtB* phytoene synthase, *crtI* phytoene desaturase.

**Fig. S2** Chromatogram of reference standards for 20 min measured at 472and 285 nm, respectively

3:

**Additional File 3: Fig. S3** Effect of the carbon source (A. 40 mM glycerol and B. 20 mM glucose) on cell growth by metabolically engineered *E. col* strains: BL21 LF (▲), BL21LG. (♦), BL21L and (●) K12L, in 50 ml batch cultures.


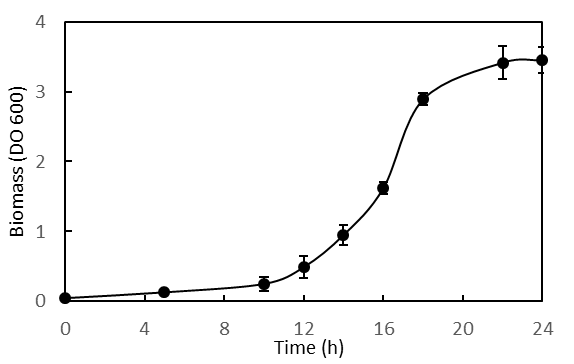


**Fig. S4** *E. coli* BL21 LF growth in the presence of octane during 24 h.

**References**

Sander LC, Sharpless KE, Craft NE, Wise S a (1994) Development of engineered stationary phases for the separation of carotenoid isomers. Anal Chem 66:1667–1674. doi: 10.1021/ac00082a012

Schierle J, Bretzel W, Bühler I, Faccin N, Hess D, Steiner K, Schüep W (1997) Content and isomeric ratio of lycopene in food and human blood plasma. Food Chem 59:459–465. doi: 10.1016/S0308-8146(96)00177-X
